# Supplementary material for: Endogenous opioids regulate social threat learning in humans
Source: Nat Commun. 2017 May 25;8:15495. doi: 10.1038/ncomms15495 (PMC5458514; doi:10.1038/ncomms15495)
Supplement: Supplementary Information — Supplementary Figures, Supplementary Tables, Supplementary Notes, Supplementary Methods and Supplementary References [file ncomms15495-s1.pdf]

Supplementary Information

Supplementary Figures

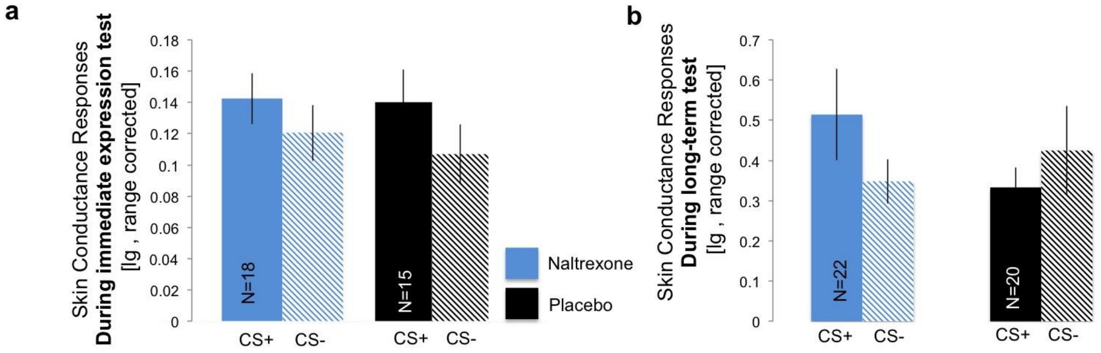

Supplementary Figure 1

(**a**) Skin conductance responses during the immediate expression test, illustrating a main effect of stimulus-type (CS+>CS-) in absence of an effect of group. **(b)** Skin conductance responses during long-term test, illustrating a stimulus-type (CS+>CS-) by group interaction, reflecting higher discrimination between CSs in the Naltrexone as compared to the Placebo group.

Error-bars denote the standard error of the mean.

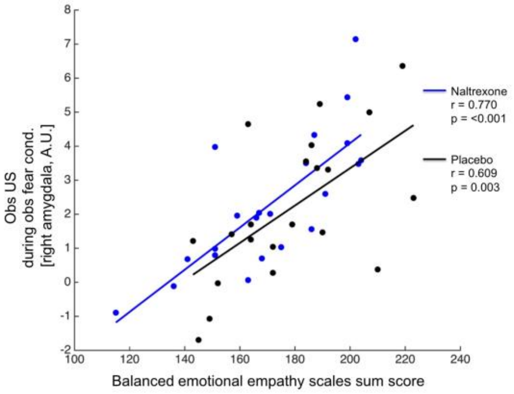

Supplementary Figure 2

Correlation between the balanced emotional empathy scales and amygdala activity towards the observational US in both groups.

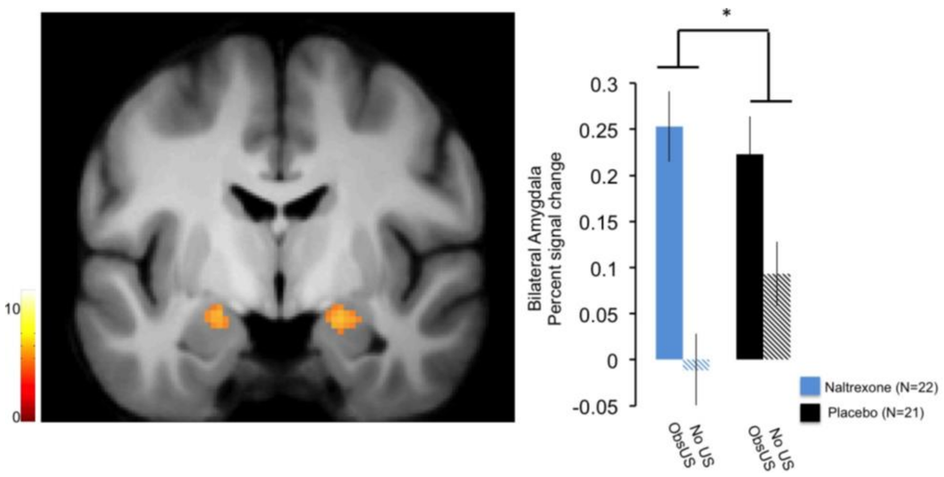

Supplementary Figure 3
Specific responses towards observational US and no observational US trials.
Amygdala responses to the observational US were enhanced in the Naltrexone group as compared to Placebo controls. The error-bars denote the standard error of the mean and T-maps are superimposed on an average structural image with a threshold of p(FWE, whole brain)<0.05 for illustrative purposes.

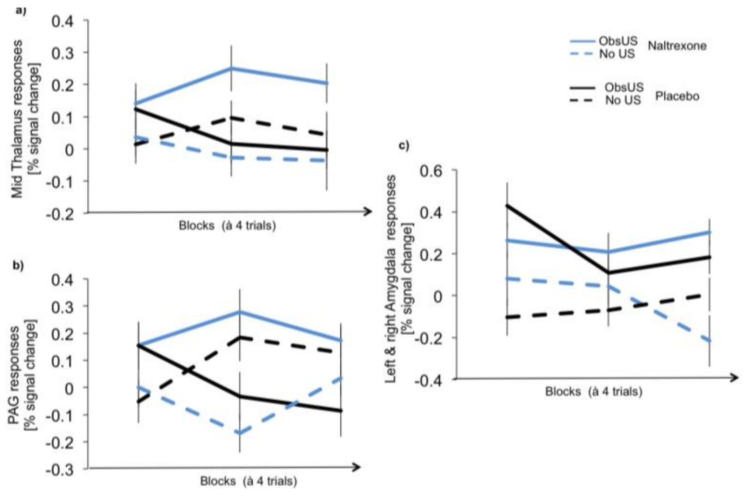

Supplementary Figure 4
Block-wise responses in the PAG and midline thalamus to the observational US were persistent over time in the Naltrexone group as compared to Placebo controls. Importantly, both groups differentiate between observational US and no observational US trails in these structures in the first block.
The error-bars denote the standard error of the mean.

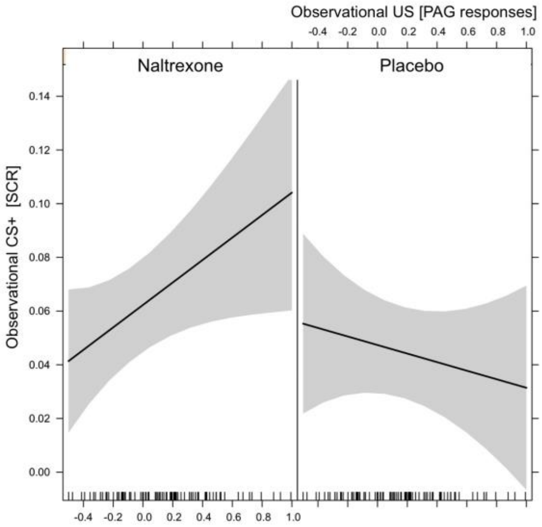

Supplementary Figure 5
Logistic linear mixed model regression of PAG responses towards the observational US predicting the SCRs to the CS+ in the Naltrexone (left) and Placebo group (right)

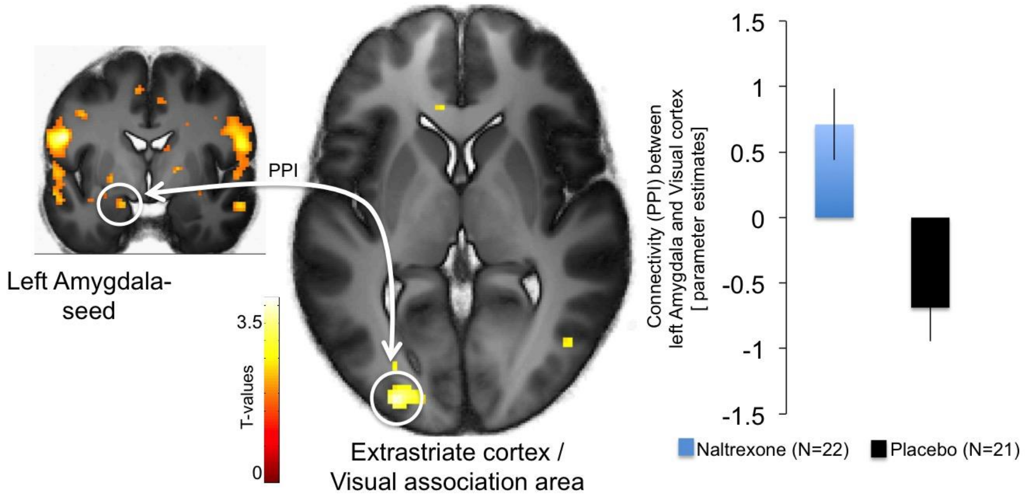

Supplementary Figure 6
Responses in the left amygdala displayed an increased functional connectivity (PPI) with in the extrastriate cortex/visual association area (Brodmann are 19) in the Naltrexone, as compared to Placebo, group [x,y,z(MNI)= x;y;z:-28;-90;2); t=3.73; p(uncorrected)<0.001]. The error-bars denote the standard error of the mean, and T-maps are superimposed on an average structural image with a threshold of p(uncorrected)<0.01 for illustrative purposes.

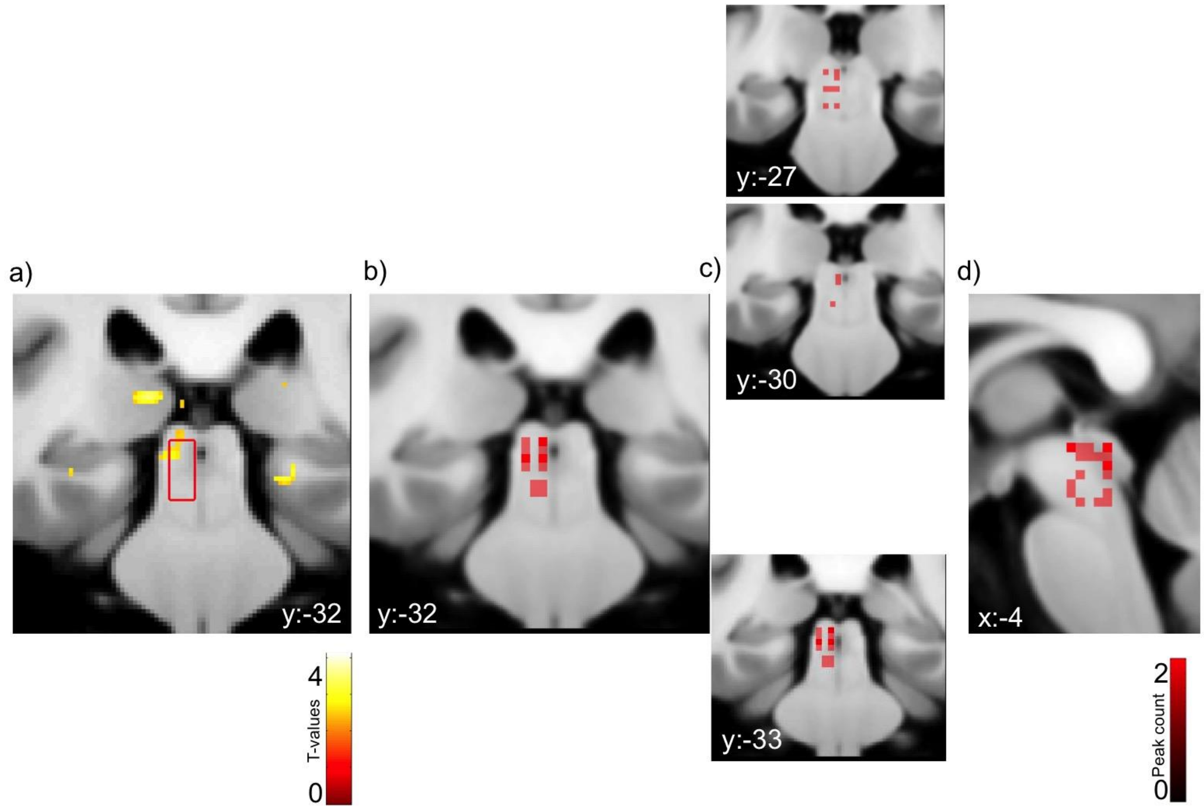

Supplementary Figure 7

Higher Responses to the observational US (obs US > no obs US) in the Naltrexone group as compared to Placebo. a) The average group difference is located within an average location (+/- SD) of the left PAG as defined in a Metaanalysis by Linnmann et al. 2012 (indicated by the red line), p(SVC)=0.026; t=3.55; x:-7;y:-32;z:-8. b-d) Location of the maxima of individual effect sizes (each square represents a participant) within this average PAG location revealed majorly activity close to the central aqueduct, and some maximal effects in neighbouring regions

Supplementary Tables

Supplementary Table 1

Results of the repeated measurements ANOVAs of the immediate test stage. Significant effects and trends (p<0.1) are marked in bold.

SCR Immediate Test stage				
	df, df error	F	p	eta^2
CS-type	**1,31**	**5.215**	**.029**	**.144**
Block	**2,62**	**44.286**	**<.001**	**.588**
Group	1,31	<1	.741	.004
CS-type* Block	**1,31**	**6.329**	**.007**	**.170**
CS-type Group*	1,31	<1	.637	.007
Block Group*	1,31	<1	.981	<.001
CS-type Block* Group*	1,31	<1	.691	.009

Supplementary Table 2

Results of the repeated measurements ANOVAs of the long-term test stage. Significant effects and trends (p<0.1) are marked in bold.

SCR long-term Test stage				
	df, df error	F	p	eta^2
CS-type	1,40	<1	.249	.006
Group	1,40	<1	.600	.007
CS-type*Group	1,40	**3.713**	**.061**	**.085**

Supplementary Table 3

Linear mixed model Regression of the SCRs towards the observational CS+

Analysis of Deviance Table (Type III Wald F tests with Kenward-Roger degrees of freedom)

Factor	F-Value	Df	Df (error)	p
**Intercept**	50.35	1	30	7.098e-08
**PAG responses towards the obs US**	4.10	1	94	0.045
**pharmacological group**	1.40	1	33	0.244
**INTERACTION** PAG responses towards the obs US * pharmacological group	3.65	1	94	0.059

Supplementary Table 4

As a control analysis, the PAG responses to the observational US did not predict the SCRs towards the observational CS-. Linear mixed model Regression of the SCRs towards the observational CS+

Analysis of Deviance Table (Type III Wald F tests with Kenward-Roger degrees of freedom)

**Factor**	**F-Value**	**Df**	**Df (error)**	**p**
**Intercept**	37.53	1	30	1.028e-06
**PAG responses towards the obs US**	1.06	1	94	0.306
**pharmacological group**	0.11	1	33	0.739
**INTERACTION** PAG responses towards the obs US * pharmacological group	2.00	1	94	0.161

Supplementary Table 5

Observational learning stage, observational US responses across groups; p(FWE, whole brain)

Contrast	Region	x	y	z	t	k	p(FWE, whole brain)
CS+ outcomes: observational US > no observational US **across groups**							
	right middle temporal gyrus	54	-62	4	11.02	2020	<0.001
	left middle temporal gyrus	-58	-52	10	7.92	677	<0.001
	right fusiform gyrus	44	-46	-20	7.89	138	<0.001
	right occipital inferior gyrus	28	-92	-2	7.34	81	<0.001
	right inferior frontal gyrus, triangular part / right anterior insula cortex	52	24	2	6.69	148	<0.001
	right amygdala	20	-4	-16	6.68	88	<0.001
	right precentral gyrus	44	4	46	6.40	73	<0.001
	NA /posterior cingulate	0	-26	24	6.36	66	<0.001
	left supramarginal gyrus	-60	-42	28	6.27	62	<0.001
	left amygdala	-20	-6	-14	6.23	20	<0.001
	right inferior frontal gyrus, opercular part	48	18	26	6.13	49	0.002
	left fusiform gyrus	-44	-54	-22	6.13	18	0.002
	cerebllum	-22	-78	-38	6.05	22	0.002

	right inferior frontal gyrus, opercular part	34	10	30	5.95	14	0.004
	right inferior frontal gyrus, opercular part	50	20	14	5.85	18	0.005
	left caudate	-10	4	8	5.53	9	0.017
	right inferior frontal gyrus, opercular part	44	26	-8	5.49	4	0.020
	left precuneus	-6	-64	34	5.36	3	0.031
	right fusiform gyrus	38	-48	-12	5.24	1	0.047

Supplementary Table 6

Observational learning stage, observational US responses between groups; p(uncorrected) < 0.001

Contrast	Region	x	y	z	t	p(uncorr)
Obs US > no US in Naltrexone > Placebo						
	Rolandic Oper L	-54	-8	24	5.53	1.85E-07
	Heschl R	58	-12	10	4.34	2.04E-05
	Rolandic Oper R	54	-8	26	4.30	2.32E-05
	Parietal Inf L	-52	-46	52	4.30	2.34E-05
	Cingulate Mid R	10	-50	28	4.23	3.01E-05
	Cerebelum 6 R	24	-54	-24	4.18	3.57E-05
	ParaHippocampal L	-34	-30	-12	4.13	4.36E-05
	Frontal Sup 2 R	22	22	56	4.12	4.57E-05
	Insula L	-36	-14	4	4.11	4.71E-05
	Temporal Sup L	-38	-58	26	4.10	4.88E-05
	Occipital Mid L	-36	-66	10	4.09	5.04E-05
	Cerebelum 6 R	16	-64	-22	4.08	5.17E-05
	Temporal Pole Sup L	-44	16	-22	4.08	5.18E-05
	Parietal Inf L	-46	-28	52	4.06	5.63E-05
	Precuneus L	-10	-48	14	4.03	6.18E-05
	Frontal Sup 2 L	-34	14	48	3.88	0.000103566
	OFCpost R	42	24	-18	3.87	0.00010796
	Postcentral L	-44	-6	38	3.86	0.000111387
	Vermis 3	0	-50	0	3.82	0.000127271
	Frontal Mid 2 L	-26	28	40	3.82	0.000127811
	Postcentral L	-60	-20	34	3.81	0.000132562
	Hippocampus R	38	-10	-12	3.80	0.000138812
	Cingulate Mid R	8	40	30	3.80	0.000140107
	Cingulate Ant R	0	46	28	3.77	0.000152183
	Cerebelum 6 L	-12	-64	-18	3.75	0.000165559
	Angular L	-40	-72	44	3.71	0.00018652
	Angular L	-50	-72	26	3.68	0.000207424
	Cerebelum Crus2 L	-28	-82	-34	3.64	0.000235927
	Precentral R	64	2	26	3.63	0.000242604
	OFClat L	-52	30	-12	3.62	0.000255647
	Cingulate Ant R	16	40	6	3.59	0.000280718

	Temporal Mid R	60	-24	-8	3.59	0.000283122
	Cingulate Ant R	10	28	16	3.57	0.000303875
	Frontal Sup Medial L	-14	60	6	3.56	0.000306576
	Temporal Mid R	54	-62	12	3.55	0.000316263
	Frontal Mid 2 R	28	16	42	3.55	0.000321818
	Temporal Mid L	-62	-16	-12	3.55	0.000322708
	Cerebelum Crus2 R	28	-78	-30	3.53	0.000343337
	Precuneus L	0	-44	38	3.52	0.000350497
	Thalamus L	-6	-26	2	3.48	0.000405595
	Angular L	-56	-58	34	3.48	0.000407141
	Calcarine L	-20	-86	0	3.47	0.000417014
	Temporal Mid R	58	-4	-20	3.46	0.000431158
	Frontal Mid 2 R	26	22	34	3.41	0.000499984
	Temporal Sup L	-50	-14	4	3.39	0.00054433
	Supp Motor Area L	-12	-10	56	3.38	0.000564487
	Temporal Mid L	-52	-20	-12	3.35	0.000618504
	Frontal Inf Tri L	-56	16	6	3.33	0.000646248
	Frontal Med Orb R	12	44	-2	3.31	0.000683995
	Angular R	56	-62	26	3.31	0.000696963
Obs US > no US in Placebo > Naltrexone						
no voxel above threshold						

Supplementary Table 7

Observational learning stage, CS responses between groups; ROI

Contrast	Region	x,y,z	t	k	p(FWE, ROI)
CS+ > CS- in Naltrexone > Placebo					
	right amygdala	-16 -4 -18	3.21	19	0.043
CS+ > CS- in Placebo > Naltrexone					
	n.s.				

Supplementary Table 8

Immediate test stage, Direct CS responses. We analyzed BOLD contrast of linearly decreasing conditioned responses over time (as indicated by the time*stimulus type interaction in the SCR) during the Immediate test stage to test if the groups differed in their hemodynamic activity during fear expression.

Contrast	Region	x	y	z	t	k	p (FWE, ROI)
Decrease in CS+ > CS- in Placebo > Naltrexone							
	right amygdala ROI	26	-8	-14	3.20	33	0.039
	left amygdala ROI	-22	-2	-24	3.11	9	0.055
Decrease in CS+ > CS- in Naltrexone > Placebo							
	n.s.						

Supplementary Table 9

Contributed weight per region to classification analysis

**Region (Harvard-Oxford Atlas)**	**ROI weight**	**Voxel in ROI**	**Exp. Ranking**
Superior Temporal Gyrus, anterior division Right	3.6824	7	0.9767
Temporal Pole Left	2.5900	82	2.4419
Caudate Right	2.5628	64	2.8372
Middle Temporal Gyrus, anterior division Left	2.3999	39	3.8140
Thalamus Right	2.2302	62	4.8140
Central Opercular Cortex Right	2.0318	47	6.4186
Occipital Fusiform Gyrus Left	1.9318	31	7.5349
Cingulate Gyrus, posterior division	1.7749	3	9.9767
Supramarginal Gyrus, anterior division Left	1.6627	54	11.2093
Temporal Occipital Fusiform Cortex Left	1.6534	260	11.1163
Caudate Left	1.6450	19	11.8605
Middle Temporal Gyrus, temporooccipital part Left	1.5976	787	12.3721
Inferior Frontal Gyrus, pars triangularis Left	1.5615	313	13.7907
Inferior Frontal Gyrus, pars opercularis Right	1.5580	65	14.0698
Lateral Occipital Cortex, inferior division Right	1.5432	135	14.7442
Cerebelum Crus2 Left	1.5158	375	16.0
Occipital Pole Left	1.5082	141	16.2093
Supplementary Motor Cortex Right	1.4780	1	19.8140
Cerebelum 7b Left	1.4399	123	19.8140
Insular Cortex Left	1.4331	124	19.8372
Middle Temporal Gyrus, temporooccipital part Right	1.3477	50	23.3721
Middle Temporal Gyrus, posterior division Left	1.3375	386	24.8140
Superior Frontal Gyrus Right	1.3357	60	25.1628

Pallidum Right	1.3176	21	27.3256
Frontal Pole Left	1.3159	179	26.8372
Cuneal Cortex Right	1.3136	175	26.3256
Frontal Orbital Cortex Left	1.3096	389	26.6047
Inferior Frontal Gyrus, pars opercularis Left	1.3089	491	26.5814
Cerebelum Crus1 Right	1.2980	539	27.9535
Cerebelum 3 Left	1.2744	147	31.3023
Lateral Occipital Cortex, superoir division Left	1.2674	66	31.1860
Vermis 6	1.2661	3	32.0698
Superior Temporal Gyrus, anterior division Left	1.2577	53	31.6744
Angular Gyrus Right	1.2435	491	31.8140
Parahippocampal Gyrus, anterior division Right	1.2082	100	34.6279
Angular Gyrus Left	1.2044	477	35.3953
Parietal Operculum Cortex Right	1.1876	8	37.3488
Lateral Occipital Cortex, superoir division Right	1.1708	272	37.4419
Temporal Occipital Fusiform Cortex Right	1.1655	27	38.2093
Middle Frontal Gyrus Left	1.1571	556	38.5581
Supramarginal Gyrus, posterior division Left	1.1464	583	39
Precuneous Cortex	1.1325	268	40.3488
Middle Frontal Gyrus Right	1.1223	27	41.5349
Inferior Temporal Gyrus, temporooccipital part Left	1.1029	68	43
Putamen Left	1.0933	8	43.9767
Lateral Occipital Cortex, inferior division Left	1.0713	1108	44.8605
Superior Frontal Gyrus Left	1.0464	233	46.3023
Insular Cortex Right	1.0404	47	46.7209
Frontal Pole Right	1.0296	2468	48.2558
Supramarginal Gyrus, posterior division Right	1.0205	103	48.3023
Parietal Operculum Cortex Left	1.0144	38	49.7674
Cerebelum Crus2 Right	1.0045	289	50.4651
Putamen Right	0.9715	64	51.6977
PP r (Planum Polare Right)	0.9428	48	54.4884
Temporal Fusiform Cortex, anterior division Left	0.9409	1	54.3721
Superior Temporal Gyrus, posterior division Left	0.9372	168	55.3488
Intracalcarine Cortex Right	0.9275	749	55.5814
Precentral Gyrus Right	0.9203	60	54.2791
Frontal Operculum Cortex Right	0.9052	27	56.2326
Central Opercular Cortex	0.8902	10	57.1395
Hippocampus Left	0.8758	118	58.8372
Accumbens Right	0.8712	155	60.3256
Inferior Temporal Gyrus, anterior division Right	0.8555	501	60.3488
Amygdala Left	0.8455	265	61.5349
Precentral Gyrus Left	0.8189	206	62.2558
Intracalcarine Cortex Left	0.7688	33	62.6047
Pallidum Left	0.7609	3	64.7209
Planum Temporale Left	0.7345	27	67.1163
Occipital Fusiform Gyrus Right	0.7320	134	66.8372
Thalamus Left	0.7152	9	68.6512
Frontal Operculum Cortex Left	0.6709	111	70.2558
Middle Temporal Gyrus, anterior division Right	0.6526	2	68.3953
Cerebelum 7b Right	0.6447	1	72.0465
Paracingulate Gyrus Left	0.6185	5	69.8140
Postcentral Gyrus Right	0.5969	73	73.4651
Cerebelum 6 Right	0.5828	123	74.1395
Amygdala Right	0.5807	30	74.5581
Middle Temporal Gyrus, posterior division Right	0.5447	2	76
Temporal Pole Right	0.5354	70	76.2326
Inferior Temporal Gyrus, temporooccipital part Right	0.5316	8	77.8372
Cerebelum Crus1 Left	0.5065	6	76.5349
Frontal Medial Cortex	0.4824	20	76.8372
Cingulate Gyrus, anterior division	0.4477	29	80.1395
Inferior Temporal Gyrus, anterior division Left	0.4366	3	78.2326
Inferior Temporal Gyrus, posterior division Right	0.3591	4	82.6047
Temporal Fusiform Cortex, posterior division Left	0.2367	6	84.2558
Lingual Gyrus Left	0.2348	1	78.9767

Cerebelum 8 Right	0.0114	1	85.4419

Supplementary Note 1: Subjective ratings

In order to control for difference in the perceived unpleasantness of the observational US, subjective ratings were compared between groups without revealing a difference ($t(38)<1$; $p>.9$). While this speaks against an inflation of the unpleasantness of observational US, the Naltrexone group rated the delivery of the US less unpleasant for the demonstrator ($t(39)=2.279$, $p = 0.028$). This effect might demark an isolated effect of opioid blockade on recognition of emotional responses in others, in line with a previous study showing that Naltrexone reduces the speed in identification of negative emotions in others [1]. Please note that the number of cases varies between analyses, since some specific values were missing for some participants.

Additionally, multiple regression of the balanced emotional empathy scales on hemodynamic activity towards the observational US revealed a cluster in the left amygdala in both groups.

Supplementary Note 2: Additional fMRI results

Additional temporal modelling of PAG responses towards the observational US

In addition to the analyses of block wise responses, we modelled exponentially decreasing responses towards the observational US (contrast obs US > no obs US) in the PAG over trials. This analysis revealed that a cluster in the PAG decreased in the Placebo group (but not the Naltrexone group). This cluster overlapped with the activity reported in our manuscript (4mm sphere; -8;-30;-10; t=3.17, p(SVC)=0.015).This confirms our analyses above that the PAG time-course shows indeed a quadratic interaction over blocks between groups.

Next, we set up a simplified first level that models prediction error responses, defined as the deviation between the outcome and the expected outcome, to test if the PAG follows such a time-course. This Prediction error was modelled as absolute difference between the observed outcome of CS+ trials (observational US = 1/ no obs US = 0) and the sum of previous outcomes divided through the trial-numbers (i.e. average of outcomes of previous trials). The prediction error term was added as a parametric modulator of CS+ outcomes (controlling for the general outcome, i.e. obs US and no obs US).

A one sample t-test of activity in the Placebo groups revealed significant activity in the PAG reflecting the time-course of the prediction error (overlap with PAG activity as reported in main text: 4mm sphere x;y;z: -8;-30;-10;t=3.77; p(SVC):0.030;).

Interestingly, other regions as the medial thalamus, medial PFC and the amygdala followed this time-course.

Importantly, the same region in the PAG was stronger correlated with this prediction error time-course as compared to the Naltrexone group, which did not follow a Prediction error related time-course (x;y;z: -8;-30;-10;t=3.21; p(SVC):0.020).

These exploratory results complement our results, suggesting that the time-course of PAG responses represent a learning related decrease of signalling to the observational US. Moreover, it suggests that blockade of opioid receptors prevents such diminution of responses.

Additional functional connectivity of PAG responses towards the observational US

In addition to the analyses of functional connectivity of the PAG seed region, we explored connectivity of the left amygdala (representing the difference between responses to the observational US between groups). Interestingly, we found that the Naltrexone group (as compared to Placebo) showed higher functional connectivity between the amygdala and the extrastriate cortex in the visual association area (Brodmann area 19; x;y;z:-28;-90;2); t=3.73; p(uncorrected)<0.001; see figure S6). This complements our results of higher connectivity between the PAG and the STS, reported in the main text, suggesting that the Naltrexone group shows enhanced processing of observed information during the observational US.

Supplementary methods

Skin conductance Responses (SCR) acquisition.

Skin conductance was measured by a pair of Ag-AgCL electrodes attached to the distal phalanges of the index and middle finger of the left hand. The physiological signals were amplified and recorded using a Biopac 150 System (Biopac Systems Inc, Santa Barbara, California, USA) and filtered between 0.05 and 5Hz. Phasic skin conductance responses towards each CS onset and the observational USs, were measured as the peak-to-peak amplitude (in microsiemens, µS) in the .5 to 4.5 second window following stimulus onset.

Logistic linear mixed model regression

Time course of the SCRs towards the observational CS+ was analyzed using a logistic generalized linear mixed models (GLMMs) with by-subject random intercept [2]. The model included as factors the extracted responses to the observational US in the PAG, the pharmacological group as well as the interaction of the two latter factors. The same model was employed in a control analysis to predict the time course of the SCRs towards the observational CS-. All reported main- and interaction effects of the GLMMs were evaluated with "Type III" analysis of deviance (i.e., analogous to Type III Sum of Squares ANOVA) tests based on the Wald statistic. Note that by using Type III analysis, the order of factors within the GLMM does not influence their significance.

Functional connectivity analysis

Psycho-physiological interaction (PPI, as implemented in SPM8) was employed to examine condition-specific functional connectivity between the PAG and whole brain voxel during the observational US (observational US > no observational US). Activity analyzed by extracting each participant's BOLD time-course eigenvariate within the PAG as a seed region. The

extracted BOLD time-course eigenvariate within the PAG ROI was deconvolved and multiplied with the condition specific onsets of the observational US > no observational US contrast. The product was entered as a regressor into a GLM for each participant controlling for the time-course of the BOLD signal in the PAG and the onset regressor for observational US > no observational US contrast, as nuisance regressors. Parameter estimates of the observational US-PPI were then contrasted between groups.

Supervised machine learning classification

For classification analysis of the differences in heamodynamic between the Naltrexone and Placebo group during expression of conditioned responses, we useed supervised machine learning. A support vector machine (SVM) as implemented in the Pattern Recognition for Neuroimaging Toolbox [PRoNTo [3]] for SPM 8 was used, which initially uses data of all participants but one that are classified as Naltrexone or Placebo to establish an optimal boundary that separates the two groups ("training"). In our study we used the beta estimates images of heamodynamic responses towards the CS+ during the immediate direct expression test, restricted to a kernel including activity in regions that were responsive to the observational US ($p<0.001$). The computed boundary is then used to predict which group the data from the left out participants belongs to in a blind manner. These steps are repeated, leaving out each individual (Cross validation of "leave one participants out"). Statistical significance of classification above chance was tested using permutation testing (1000 permutations) with a random assignment of group class to the beta estimate images.

Supplementary References:

1. **Wardle, M. C., Bershad, A. K. & de Wit, H. Naltrexone alters the processing of social and emotional stimuli in healthy adults. *Soc Neurosci* 1–13 (2016). doi:10.1080/17470919.2015.1136355**
2. **Baayen, R. H., Davidson, D. J. & Bates, D. M. Mixed-effects modeling with crossed random effects for subjects and items. *Journal of Memory and Language* 59, 390–412 (2008).**
3. **Schrouff, J. *et al*. PRoNTo: pattern recognition for neuroimaging toolbox. *Neuroinformatics* 11, 319–337 (2013).**
